# Luminescence of delafossite-type CuAlO_2_ fibers with Eu substitution for Al cations

**DOI:** 10.1080/14686996.2016.1172024

**Published:** 2016-04-25

**Authors:** Yin Liu, Yuxuan Gong, Nathan P. Mellott, Bu Wang, Haitao Ye, Yiquan Wu

**Affiliations:** ^a^ New York State College of Ceramics, Alfred University, Alfred, NY, USA; ^b^ School of Engineering and Applied Science, Aston University, Birmingham, UK

**Keywords:** CuAlO_2_, electrospinning, luminescent materials, 40 Optical, magnetic and electronic device materials, 105 Low-Dimension (1D/2D) materials, 100 Materials, 204 Optics / Optical applications, 200 Applications

## Abstract

CuAlO_2_ has been examined as a potential luminescent material by substituting Eu for Al cations in the delafossite structure. CuAlO_2_:Eu^3+^ nanofibers have been prepared via electrospinning for the ease of mitigating synthesis requirements and for future optoelectronics and emerging applications. Single-phase CuAlO_2_ fibers could be obtained at a temperature of 1100 °C in air. The Eu was successfully doped in the delafossite structure and two strong emission bands at ~405 and 610 nm were observed in the photoluminescence spectra. These bands are due to the intrinsic near-band-edge transition of CuAlO_2_ and the f-f transition of the Eu^3+^ activator, respectively. Further electrical characterization indicated that these fibers exhibit semiconducting behavior and the introduction of Eu could act as band-edge modifiers, thus changing the thermal activation energies. In light of this study, CuAlO_2_:Eu^3+^ fibers with both strong photoluminescence and p-type conductivity could be produced by tailoring the rare earth doping concentrations.

## Graphical abstract

**Figure:**
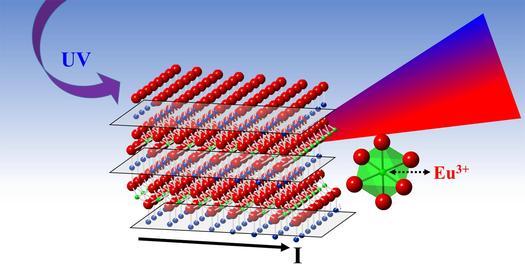

## 1. Introduction

Delafossite-type CMO_2_ ternary oxides (C represents monovalent ion and M represents the trivalent ion) have been widely studied as p-type transparent conducting oxides and exhibit promise for various optoelectronics applications.[1–5] This class of materials has a rhombohedral space group of R$\overset{¯}{3}$m. The c-direction of the layered delafossite is occupied by a linearly coordinated O-Cu-O dumbbell. The edge-sharing MO_6_ octahedra constitute the MO_2_ layer along the *ab* plane, which is connected by the Cu^+^ ions. CuAlO_2_ is a typical *p*-type transparent oxide with a wide band-gap (>3 eV) and room temperature photoluminescence through its UV near-band-edge emission due to the recombination of free excitons.[1,6,7] The delafossite structure allows for chemical bond stretching from either the Cu-O bonds (*xy*-plane) or the Al-O bonds (*z*-direction) and the local lattice relaxations are crucial to electrical properties. By substituting the Al cation site with other trivalent ions, the conductivity could be enhanced due to the change of the electronic density via ligand field modification.[8,9]

While most researchers focus on the electrical properties of CuAlO_2_ as a p-type transparent conducting oxide, there are few reports of CuAlO_2_ as a potential phosphor material. In addition, with the development of field emission display and other flat display technologies, new generation of phosphors with good luminescence, conductivity and stability are required. CuAlO_2_ could be a promising host material in which the Al site could be substituted with various trivalent rare earth dopants, without changing the hole transport within the Cu^+^ plane.[9] Since the main conduction path in delafossite crystals is close-packed Cu^+^ layers,[10] the electrical properties could be sustained in addition to photoluminescence properties. Conventional solid-state synthesis of CuAlO_2_ powders or thin films requires high temperature sintering and repeated thermal treatments. In this study, we prepared CuAlO_2_ fibers via a cost-effective electrospinning method. The wire-like CuAlO_2_ nanostructures possess higher surface area and sintering activity, which could lower the annealing temperature. Additionally, the one-dimensional material may also present extraordinary effectiveness in light emitting and transparent conducting.[11–13] The dopant cation, Eu^3+^, was chosen as the emission activator and intense red emission from f-f transition of Eu^3+^ was identified. The Eu^3+^ activator center was successfully doped into the Al^3+^ site and this delafossite-type material could be used as potential host for luminescence application.

## 2. Experimental details

The 0.01 mol CuAl_1-x_Eu_x_O_2_ (x = 0.001, 0.003, 0.01, 0.03, 0.05 and 0.1) was prepared through stoichiometric mixing of copper nitrate, aluminum nitrate and europium nitrate in deionized water. Poly(vinylpyrrolidone) (PVP, molar weight ~140,000 g mol^–1^) was then added into the aqueous solution to obtain desirable viscosity for electrospinning. The viscous solution was then electrospun onto aluminum foil via a self-made electrospinning stage. The as-spun polymer fibers were collected and annealed in air at 1100 °C for 3 h.

The thermo-gravimetric (TG) and differential thermal analyses (DTA) were carried out on Q600 TG/DTA (TA Instruments, New Castle, DE) in flowing air. The powder X-ray diffraction measurement was performed on Bruker D2 Phaser (Bruker-AXS, Madison, WI) using Cu Kα radiation line. The Rietveld refinement was carried out by TOPAS software kits (Bruker-AXS, Madison, WI). The microstructure of the electrospun fibers was observed through an FEI Quanta 200F environmental scanning electron microscope (ESEM) (Fei, HIllsboro, OR) combined with energy dispersive spectroscopy (EDS). Steady state photoluminescence (PL) spectrum measurements were performed on a Spex FluoroLog Tau-3 (HORIBA Jobin Yvon, Edison, NJ) at 300 K. A Fourier-transform Raman spectrometer (Thermo Nicolet 6700, Thermo Nicolet Corp., Madison, WI) was used to record room-temperature Raman spectra of the delafossite samples. X-ray photoelectron spectroscopy (XPS) (PHI Quantera, ULVAC-PHI, Chanhassen, MN) was used to examine the local chemical environment in the doped samples (x = 0.001, 0.003, 0.01). The X-ray source was a monochromated Al Kα line at 1486.6 eV. The beam sweeps for each high resolution scan were adjusted (three sweeps: Al_2p_/Cu_2p_; one sweep: O_1s_ and C_1s_) to yield a signal-to-noise ratio of >100:1 with exception of doping agents Eu_4d_ (three sweeps), which were adjusted to yield a signal-to-noise ratio of >50:1. The Hall measurement (MMR Technologies, San Jose, CA) was conducted at room temperature to determine the carrier type and density. The fibrous mat was cold pressed onto a square glass substrate and coated with silver electrodes on the four corners.

## 3. Results and discussion

**Figure 1.**
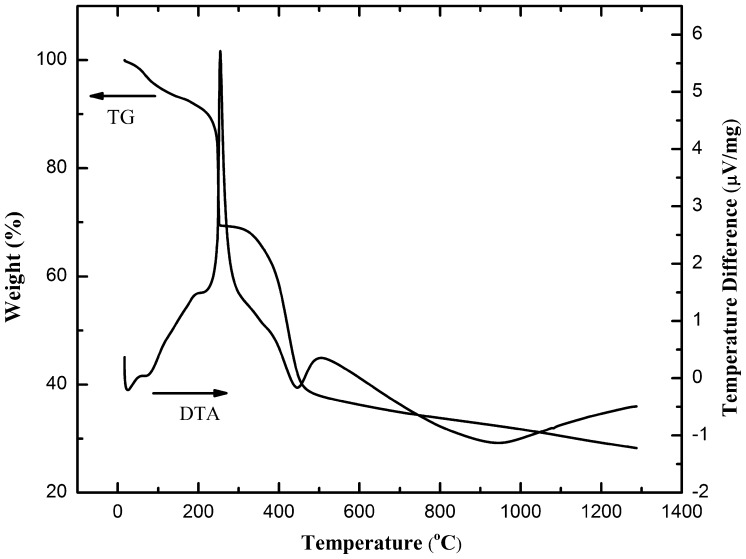
TG-DTA curve of as-spun polymeric fibers.

Thermal treatment of electrospun fibers involves the burnout of organics and simultaneous crystallization. The thermo-gravimetric and differential thermal analyses are shown in Figure 1. The TG curve consists of three stages of weight loss. The first weight loss event occurred at ~100 °C, which could be attributed to the volatilization of adsorbed water and surface organics. The second event, at ~225 °C, is accompanied by a strong thermopositive peak in the DTA signal. This is due to the decomposition of PVP lateral chains and the nitrate salts. The third stage of weight loss begins at 400 °C, with a small thermal positive peak, which could correspond to the further burnout of PVP fundamental chains.[14,15] At above 800 °C, the TG curve exhibits a broad endothermic peak in the DTA curve. The TG plateau indicates that the thermal decomposition process was complete and the decomposed copper and aluminum nitrate undertook a combination reaction to finally form CuAlO_2_. The overall weight loss is nearly 80%, given the large amount of PVP addition into the precursors required to yield ideal elasticity for electrospinning.

**Figure 2.**
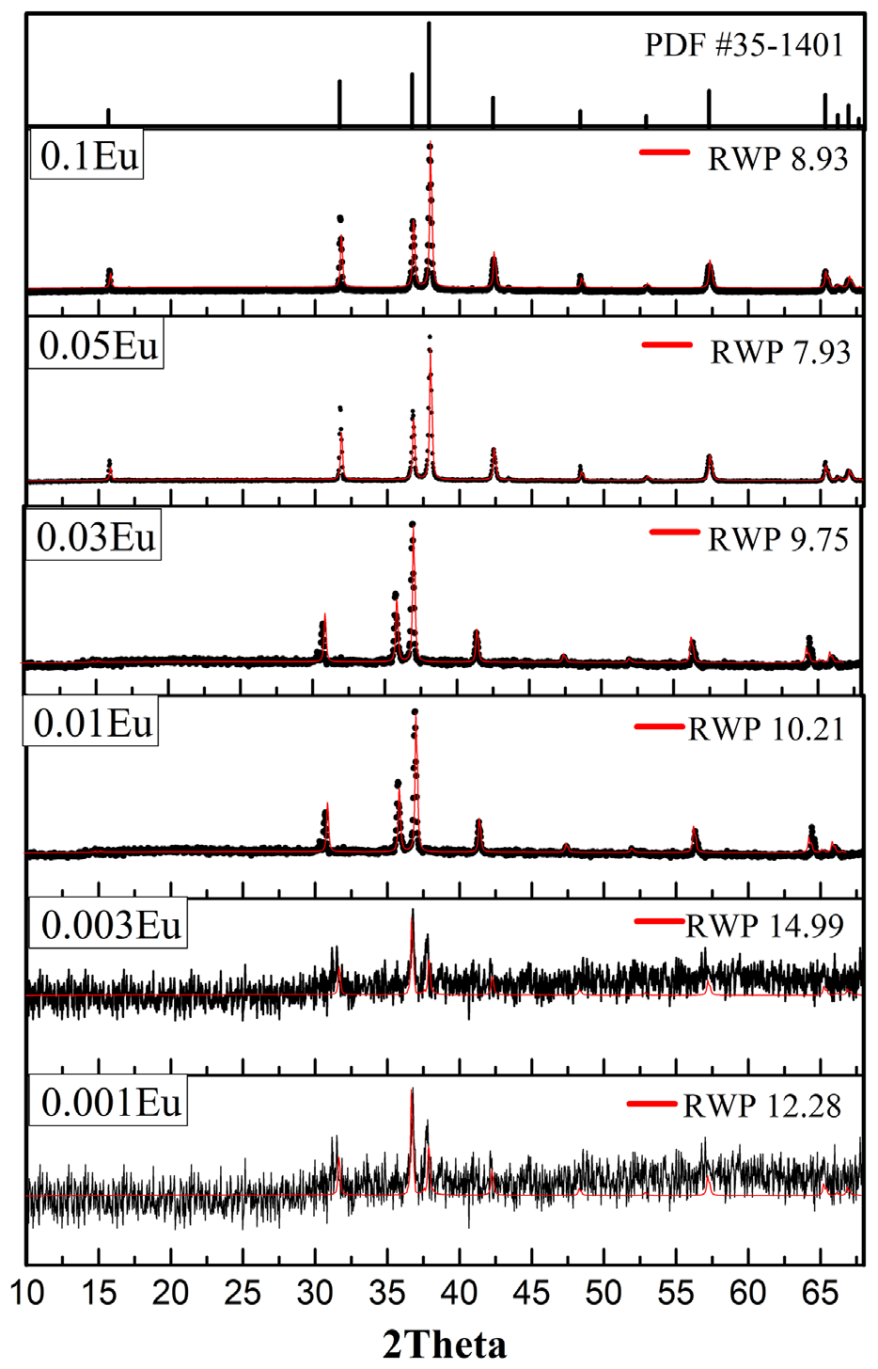
XRD patterns and profile fitting of CuAl_1-x_Eu_x_O_2_ (x=0.001, 0.003, 0.01, 0.03, 0.05, 0.1) electrospun fibers annealed at 1100 °C for 3 h, with standard PDF#35-1041 corresponding to 3R-polytype delafossite CuAlO_2_.

The X-ray diffraction (XRD) patterns together with RWP (residual weight pattern) values from the refinements are shown in Figure 2 along with the standard CuAlO_2_ (R$\overset{¯}{3}$m) phase for comparison. All samples show a single CuAlO_2_ phase with no other crystal structure identified. This shows that the post annealing condition is sufficient to transform the polymeric as-spun fibers into a single, crystalline delafossite structure. In addition, samples (not shown in Figure 2) with a higher annealing temperature of 1200 °C show undesired CuAl_2_O_4_ phase due to the oxidation of cuprous ions. Annealing at lower temperatures than 1040 °C, both CuAlO_2_ and CuO phases result, due to the insufficient reaction between CuO and CuAl_2_O_4_.[16,17] It is noteworthy that crystallinity differs among samples with different Eu doping. Samples with the 0.001 and 0.003 Eu doping show lower degree of crystallinity corresponding to broader diffraction peaks. However, when the doping concentration exceeds 0.01, well-defined CuAlO_2_ delafossite peaks are exhibited. According to the bonding environment shown later, the substituted Eu and resultant Eu-O bond may alter the bonding environment, allowing for higher mass diffusivity and more sufficient crystallization, which might explain the promotional effect of Eu on CuAlO_2_ crystallization. In summary, the nanofiber-derived single-phase CuAlO_2_ via single step annealing and relatively short dwelling time implies that one-dimensional ceramic fibers have higher sintering activity than powders or thin films.[18]

**Figure 3.**
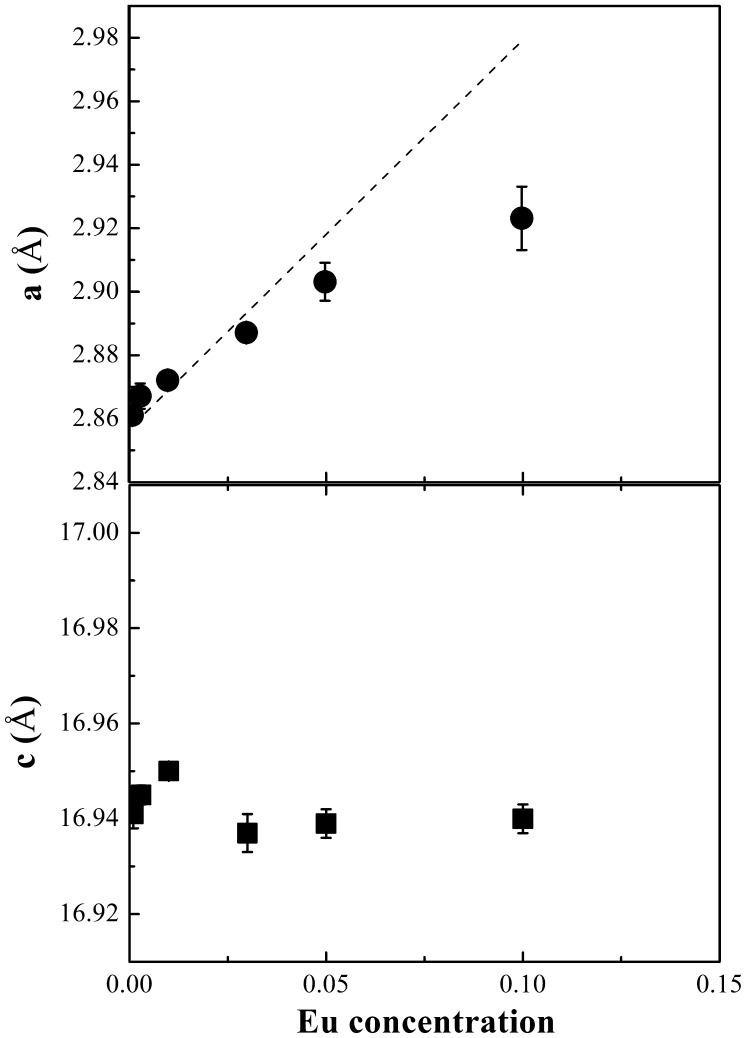
The lattice parameters *a* and *c* of the 3R-polytype structure delafossite CuAl_1-x_Eu_x_O_2_ as a function of Eu concentration. The dashed line shows the calculated a values based on *a* = 2.784 × *r*_*R*_ + 1.437, where *r*_*R*_ = (1 - *x*)*r*_*Al*_ + *xr*_*Eu*_ (Vegard’s law).

The lattice parameters extracted from the Rietveld refinement of the delafossite phase CuAl_1-x_Eu_x_O_2_ are shown in Figure 3. The lattice parameter a increases with increasing Eu concentration while c remains nearly constant. This behavior could be explained by a pseudolinear relationship between lattice constant and trivalent ion radius *r*_*R*_ in a delafossite structure.[19] According to our host material, CuAlO_2_, the lattice constant could be estimated by *a* = 2.784 × *r*_*R*_ + 1.437, in which the constant 2.784 was calculated from the *a* value at zero doping level from the standard powder diffraction file (PDF#35-1401). The trivalent ion radius *r*_*R*_ follows a weighted sum of both Al^3+^ radius and Eu^3+^ radius (*r*_*R*_ = (1 – *x*)*r*_*Al*_ + *xr*_*Eu*_).[20] By employing the Vegard’s law, the Eu^3+^ ions are assumed to exclusively occupy the Al^3+^ sites, due to their equivalent charge and more difficulties in impurity doping of Cu-O dumbbells with strong covalency between Cu^+^ d^10^ levels and O-2p orbitals. Based on the two equations above, the calculated value is shown as a dashed line in Figure 3. It can be seen that both experimental and calculated parameters follow a similar trend as a function of Eu concentration. The deviation could be attributed to the stoichiometric variation in starting precursors and the difference between nominal and doped Eu ion concentrations. The c value is independent of *r*_*R*_ and at various doping levels, the lattice parameter c remains almost constant. The trends in both *a* and *c* in terms of dopant concentration imply that Eu^3+^ substitutes Al^3+^ site in this delafossite structure.

**Figure 4.**
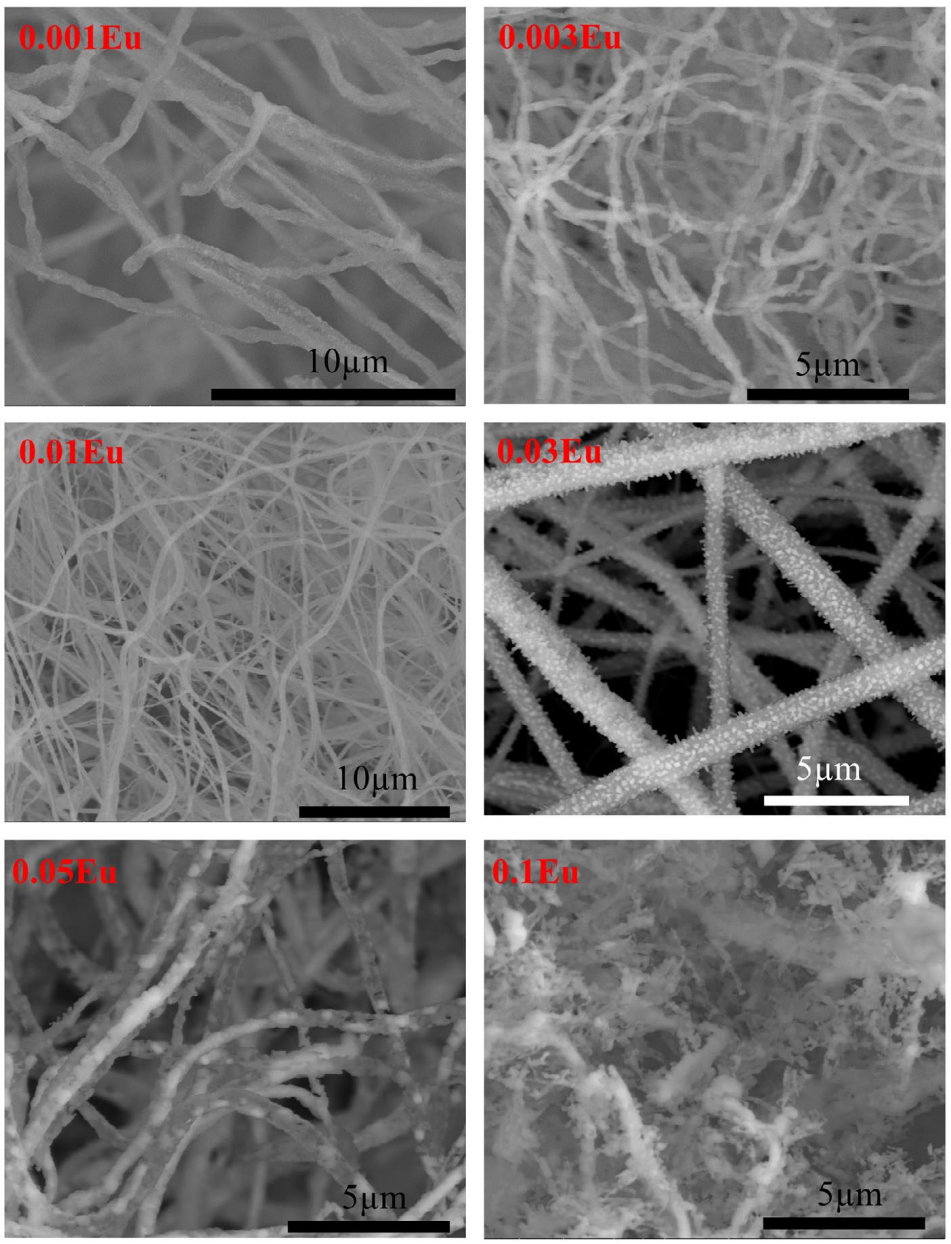
SEM microstructure of CuAl_1-x_Eu_x_O_2_ (x=0.001, 0.003, 0.01, 0.03, 0.05 and 0.1) electrospun fibers annealed at 1100 °C for 3 h.

The SEM micrographs are shown in Figure 4. After thermal annealing, the smooth polymeric as-spun fibers become coarse and rough, with nano-sized polygonal grains observed on the fibers. The microstructure evolution with the increasing doping concentration is also prominent, from well-sustained non-woven fibers to a disconnected porous structure. According to the significant radius difference between Eu^3+^ and Al^3+^ ions, the introduction of Eu^3+^ in the Al^3+^ site may cause lattice distortions and unrelaxed strains. Additionally, the radial crystallization on self-supported fibers imposes excess surface strain on the crystallites. As a result, excess enthalpy was produced for crystallization and a higher crystallinity is expected with a further increasing in Eu level, in a similar manner revealed from XRD patterns. When the Eu concentration increased with further increase in crystallinity to a degree that the bonding to crystallites cannot hold the accumulated stress in the cylindrical fibrous structure, the non-woven fibers break up and the crystallites start growing as isolated particles.

**Figure 5.**
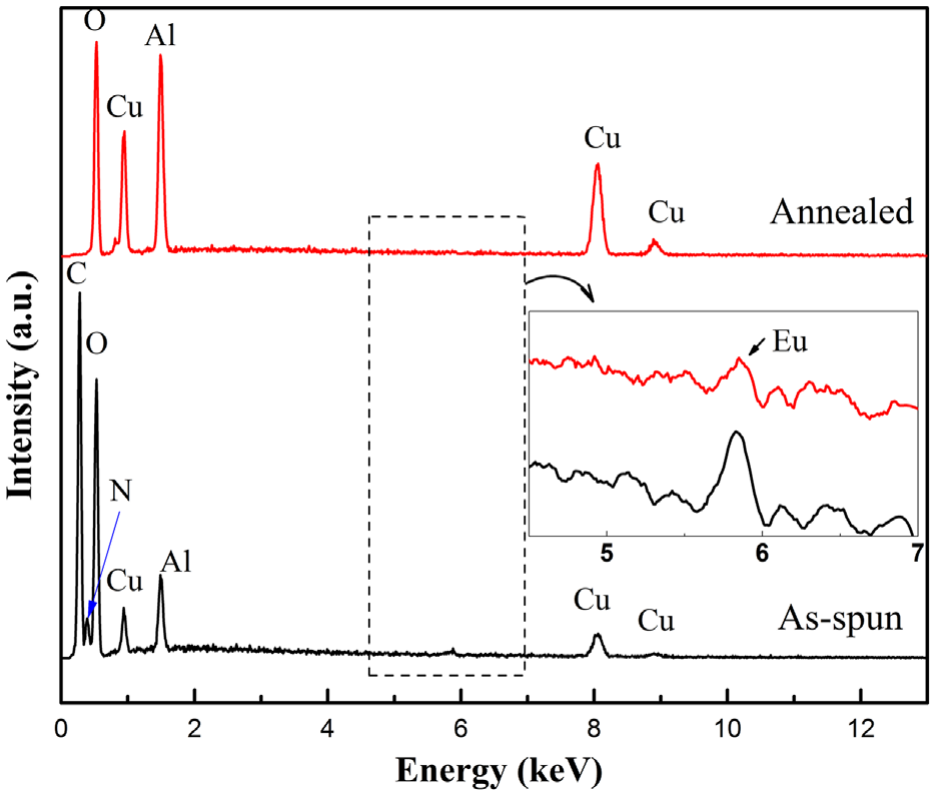
EDS spectra of as-spun polymeric fibers and annealed CuAl_0.99_Eu_0.01_O_2_ ceramic fibers. The enlarged inset shows the presence of Eu.

During the annealing process of the electrospun fibers, the organics derived from the PVP addition must be eliminated in order to achieve well-crystallized ceramic fibers suitable for luminescence application. EDS was employed to confirm the burn-out of the polymers shown in Figure 5. Before thermal annealing, the energy peaks associated with C and N were prominent because of the existence of PVP ((C_6_H_9_NO)_n_). After 1100 °C annealing, there were no C or N peaks identified; only Cu, Al, O, and Eu (enlarged inset in Figure 5) peaks were observed. The polymer burn-out, thermal decomposition and crystallization proceeded simultaneously during the annealing, which shows that this delafossite-type phosphor could be synthesized as one-dimensional fibers.

**Figure 6.**
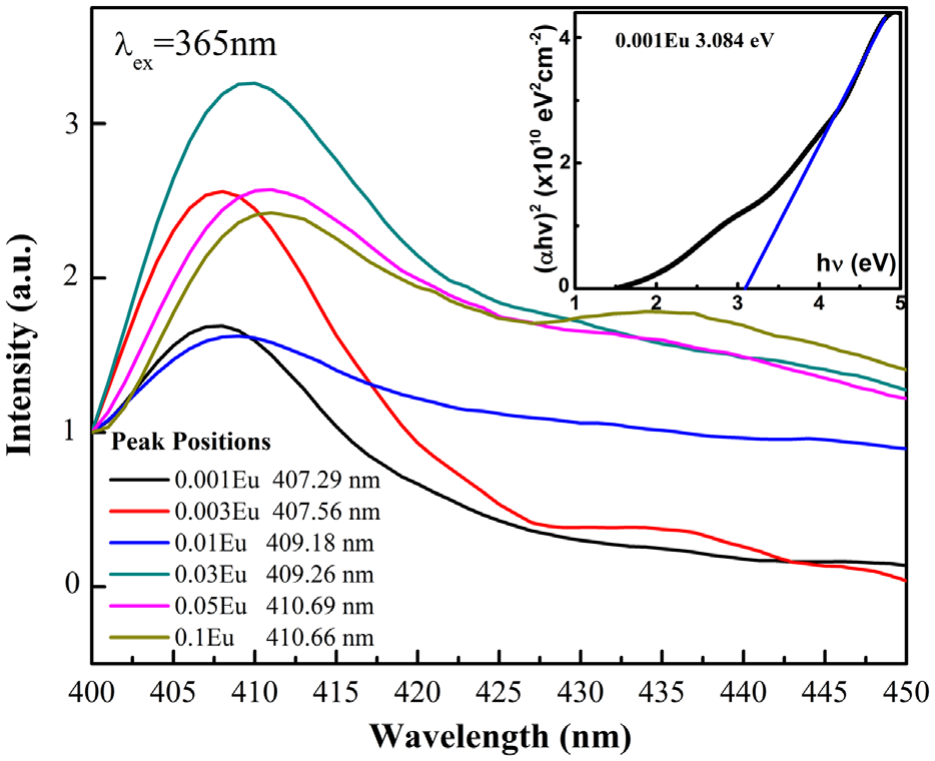
Near-band-edge photoluminescence of CuAl_1-x_Eu_x_O_2_ excited at 365 nm. Inset shows the Tauc plot used for evaluating the optical band gap of CuAl_0.999_Eu_0.001_O_2_.

The photoluminescence of CuAl_1-x_Eu_x_O_2_ (x = 0.001, 0.003, 0.01, 0.03, 0.05 and 0.1) electrospun fibers has been studied at two excitation wavelengths (365 and 420 nm). Delafossite-type CuMO_2_ has room temperature luminescence due to its UV near-band-edge emissions [21–24]. The Cu-O bonds in the O-Cu-O dumbbell layers determine the electronic structure near the band-gap and lead to strong localization of excitons in the *x*–*y* plane as well as larger binding energy. This causes hybridization between the $3{\text{d}}_{\text{z}}^{2}$ and 4s orbitals in the CuMO_2_ delafossite. Since the binding energy exceeded the room temperature thermal energy (kT ≈ 0.025 eV), room temperature PL emissions were presented.[6,7] We herein reported the room temperature near-band-edge emissions from the Eu-doped CuAlO_2_ fibers with slightly different peak energies compared to other studies.[7,25] The peak positions in terms of relative intensity shown in Figure 6 mainly centered at ~407 nm, indicating a 0.3 eV decrease in the direct band-gap compared to a normal 370 nm emission in thin films or bulks. Since the reported direct band-gap values of CuAlO_2_ vary from 3.0 to 3.8 eV and highly depend on fabrication method, the near-band-edge emission is supposed to shift accordingly. The oxygen vacancies, surface defects and crystallinity associated with fabrication methods in low dimensional CuAlO_2_ thin films or nanoparticles often yield dispersed band-gap values. In this particular study, the peak energy shift is also attributed to a fiber-derived band-gap energy change. A similar phenomenon has been observed in TiO_2_ ceramic fibers,[26] i.e. that band-gap increases with the excess pressure on the fiber circular cross section. The inset in Figure 6 shows the measured optical band gap in our study, the absorption edge (3.084 eV corresponds to ~402 nm) of which is similar to the PL peak energy, which leads to the conjecture that the emission with the excitation wavelength of 365 nm results from the CuAlO2 near-band-edge transition. We also need to consider other factors regarding to the violet emission. The defect origin from V_Cu_ in the delafossite material is assumed to form an acceptor level above the zero vibrational level,[27,28] leading to a Stokes shift in the spectrum. Since our experiments were performed at room temperature and no other peaks associated with Eu^3+^ activators were observed from the emission spectra, the broad violet emission is unlikely from transition to defect levels. Byrne et al. [25] recently observed a coexistence of both a near-band-edge UV emission and a blue emission at ~430 nm at 14.5 K, with almost 100 nm Stokes shift. Revisiting the study by Jacob et al. [29] on CuYO_2_ and CuLaO_2_, the prolific hybridization from Cu 4p/3d orbitals and the resultant $3{\text{d}}_{\text{z}}^{2}$ + 4s and $3{\text{d}}_{\text{z}}^{2}$ - 4s splitting only give rise to an asymmetric and overlapped peak feature rather than large Stokes shift. In addition, the effect of defect states on room temperature excitation still remains unclear, which definitely requires further investigation. The one-dimensional fibrous microstructure with major surface defects may also cause a band-gap narrowing in some oxide materials.[30] However, since there have been no reports on the photoluminescence of CuAlO_2_ in the form of fibers to the best of our knowledge, at the current stage we may conjecture that the decrease in the band-gap associated with lower excitation energy (365 nm in our case) leads to the red shift in the PL emission spectra. At different dopant levels, the band-gap extracted from the PL peak energies in Figure 6 decreases with the increase of Eu level, indicating the further band modifications as a result of trivalent ion substitution and change of degree in Cu 4p/3d hybridization.

We also compared the peak energies in terms of doping levels. Regardless of the variation in relative PL emission intensities, the spectrum shifts to longer wavelength as the doping level goes up, roughly from 407.29 nm in CuAl_0.999_Eu_0.001_O_2_ to 410.66 nm in CuAl_0.9_Eu_0.1_O_2_. Based on previous study,[29,31] this monotonic shift to lower energies is due to the change of hybridization between Cu 4p_x,y_ to $3{\text{d}}_{\text{z}}^{2}$ ^^± 4s, under the influence of trivalent partial substitution on Cu-O bond. As the Cu-O covalency increase from CuAlO_2_ to CuAl_1-x_Eu_x_O_2_, hybridization between the $3{\text{d}}_{\text{z}}^{2}$ and 4s orbitals became more prolific which would lift the non-bonding $3{\text{d}}_{\text{z}}^{2}$-4s orbital energy in order to compensate for the antibonding $3{\text{d}}_{\text{z}}^{2}$ + 4s orbitals. Therefore the transition energy between $3{\text{d}}_{\text{z}}^{2}$-4s and 4p_x,y_ is lowered upon introducing Eu^3+^ ions. Our observation also confirmed the prediction that this Stokes shift would increase as the size of trivalent metal ion increases.[25] For all the doped samples at excitation wavelength of 365 nm, only the broad near-band-edge emission was identified and no peaks associated with Eu f-f transitions were observed above 500 nm. At higher excitation energies, the excitons near the band edge may undergo fast recombination across the band-gap and non-radiative relaxation to the ground state while the transitions to Eu activators become absent.

**Figure 7.**
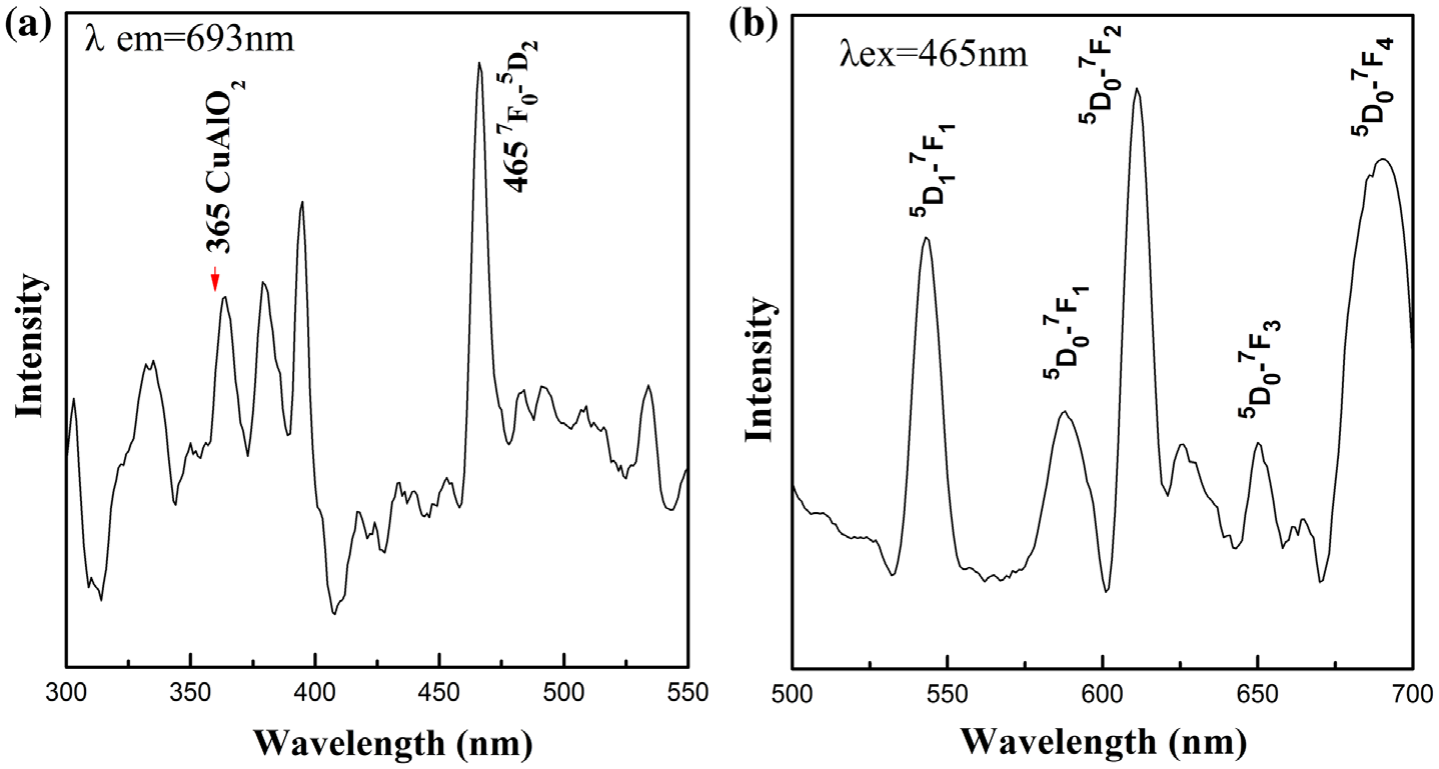
Photoluminescence excitation spectrum (a) showing the direct band-gap transition in CuAlO_2_ host, and the emission spectrum (b) of CuAl_0.99_Eu_0.01_O_2_.

**Figure 8.**
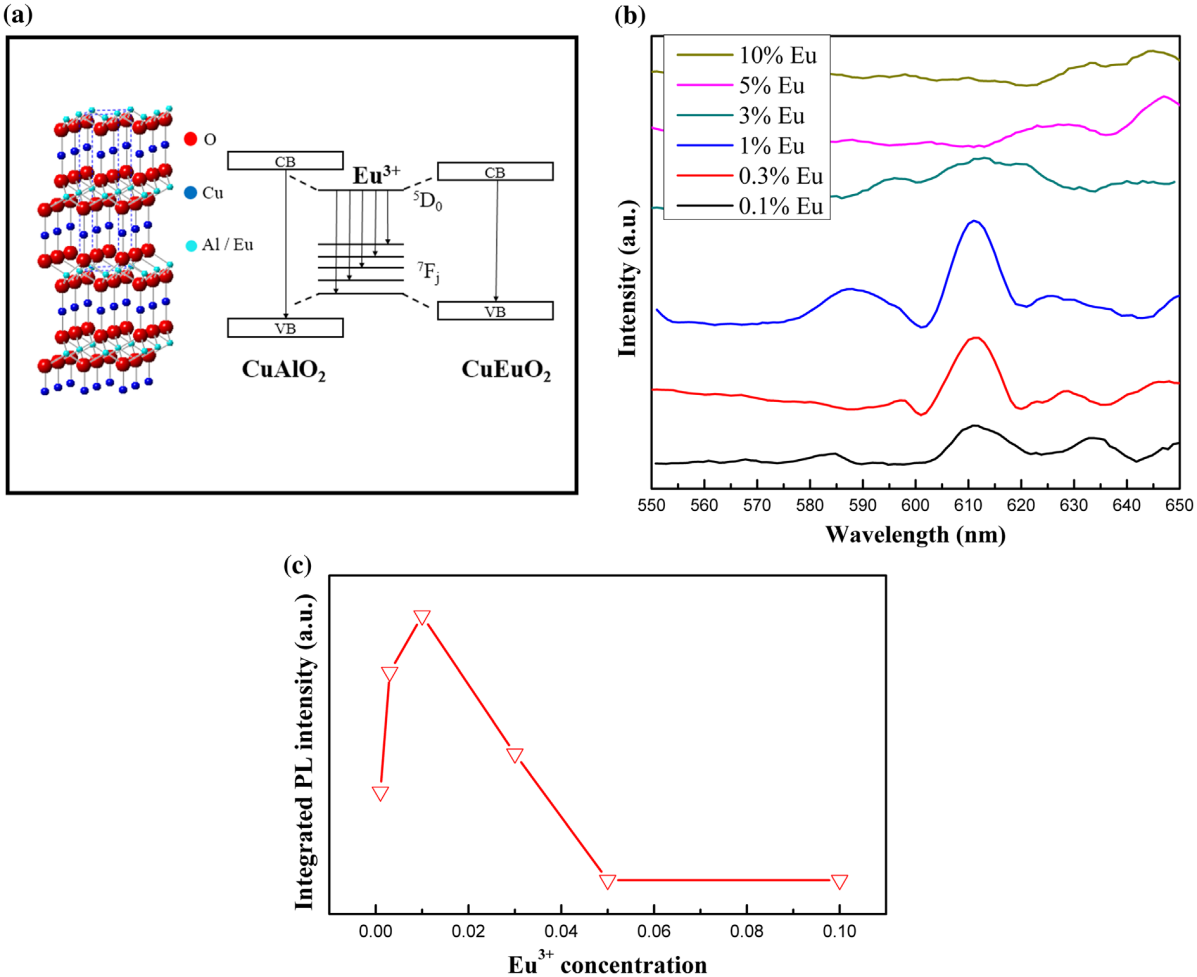
Schematic diagram of photoluminescence emission channels in the rare-earth doped delafossite CuAlO_2_ (a), ^5^D_0_ →^7^F_1_ and ^7^F_2_ transitions with respect to doping concentration (b) and integrated PL intensity of 610 nm emission as a function of doping concentration (c).

PL excitation and emission spectra from CuAl_0.99_Eu_0.01_O_2_ are shown in Figure 7. The excitation peaks between 300 and 500 nm correspond to Eu^3+^ intra-4f transitions. The ^7^F_0_-^5^D_2_ line at 465 nm is the strongest line in the excitation spectrum. The peak at 365 nm was identified as the intrinsic excitation peak from CuAlO_2_, according to Ahmad’s work on CuAlO_2_ nanoparticles [32] and our previous work on nanofibers.[31] The emission spectrum with 465 nm excitation shows broad bands at 587, 610, 654 and 690 nm, which are due to the f-f transition of ^5^D_0_ → ^7^F_J_ of Eu^3+^, respectively. For the 0.01Eu sample, ^5^D_1_ → ^7^F_1_ transition was also observed. The two-channel emission diagram is depicted in Figure 8(a). The strongest ^5^D_0_ → ^7^F_2_ red emission is the result of hypersensitive transitions with ΔJ = 2 indicating the environment around the activator centers. The increasing ^5^D_0_ → ^7^F_2_ emission intensity from samples with x = 0.001–0.01 indicates a higher symmetry local site. Upon the occupancy of Eu^3+^ ions in the octahedral center, the lattice was distorted and the local symmetry of Eu^3+^ continued to change with increasing Eu^3+^ doping level. As a consequence, the probability of each ^5^D_0_ → ^7^F_J_ transition channels may change, which leads to the variation of intensity of the six ^5^D_0_ → ^7^F_2_ emissions shown in Figure 8(b). The overall red emission intensity increases with the increase of doping concentration up to 0.01Eu. Above this doping level, the intensity significantly drops due to the concentration quench effect [33,34] shown in Figure 8(c). With the increase of Eu^3+^ concentration, the cross-relaxation becomes dominant due to the reduced distance between two Eu^3+^ activator ions. Therefore, the luminescence emission intensity almost vanishes at higher Eu^3+^ concentration. Since the activator is introduced solely on the Al^3+^ site, the critical transfer distance (*R*_*c*_) could be estimated by the following equation:

(1) $$R_{c}\approx 2{\left(\frac{3V}{4\pi x_{c}N}\right)}^{\frac{1}{3}}$$

where V is the unit cell volume, x_c_ is the critical concentration and N is the number of Al^3+^ ions per unit cell. For CuAlO_2_ host (*N* = 3), x_c_ is roughly equal to 0.01 at which concentration the maximum luminescent intensity was observed. The cell volume from XRD refinement is 120.75 Å^3^ and the R_c_ was calculated to be approximately 20 Å. For a Eu^3+^–Eu^3+^ distance larger than 5 Å, the multipolar interaction is dominant and the exchange interaction is negligible.[35] Therefore, the main mechanism for concentration quenching in the CuAlO_2_:Eu^3+^ phosphor is the multipolar interaction.

**Figure 9.**
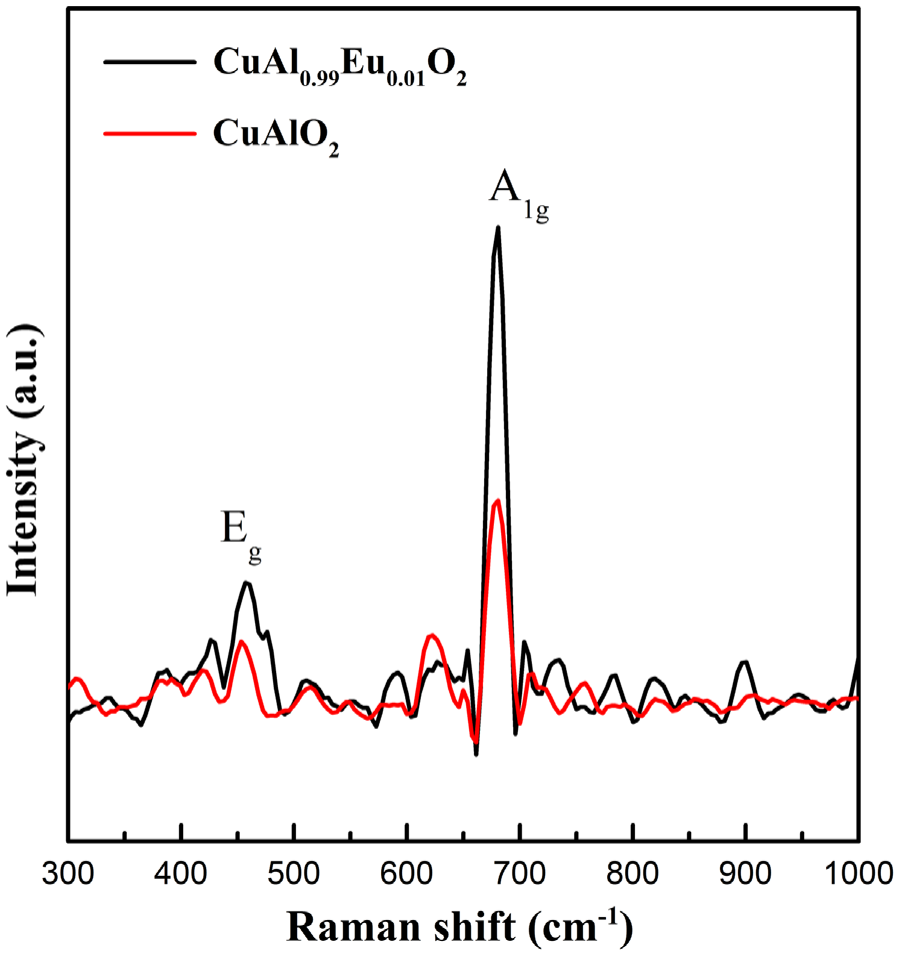
Room temperature Raman spectrum under 1064 nm excitation provided by a Nd:YVO_4_ laser.

Room temperature Raman spectra are shown in Figure 9. It has been widely acknowledged that in delafossite structure with Γ = A_1 g_+E_g_+3A_2u_+3E_u_, only the first two modes are Raman active.[36,37] The A modes correspond to the vibrational movements in the direction of Cu-O bonds and the E modes represent the vibrations in the perpendicular direction. A previous report on particulate CuAlO_2_ [25] shows two sharp peaks at 418 and 767 cm^-1^, respectively. However, the Raman scattering peaks could shift to a large extent depending on the trivalent ion species. CuLaO_2_ with the same crystal geometry has two peaks centered at 318 and 652 cm^-1^.[38] The lowered frequencies could be due to a higher M atom mass and weaker M-O bond. The room temperature Raman spectrum that we obtained here shows a similar E_g_ position but a slight lower frequency of A_1 g_ mode, which might provide supplementary information on trivalent substitution. Upon the introduction of larger Eu^3+^ ions, the MO_6_ along the *ab* plane would go through a decrease in bonding energy, which is also confirmed by XPS in later discussion. Therefore the E_g_ characterized by the vibration along the *ab*-plane mode should shift to lower frequency. However from the experimental data, the E_g_ shows a typical CuAlO_2_ peak position whereas the A_1 g_ peak shifts to lower frequency for both undoped and doped samples, which might lead to the conjecture that the decrease of the A_1 g_ frequency might due to the electrospinning synthesis during which the MO_6_ octahedral bonding energy is lowered. Another possible explanation for the decrease of A_1 g_ mode could be extrapolated from our near-band-edge emission observation. Since the Cu-O covalency is predicted to increase in the doped sample, the higher degree of hybridization might cause the A_1 g_ vibration mode to move to lower frequency. In addition, the band width of E_g_ mode becomes broader after Eu doping, indicating that the E_g_ mode is more sensitive to the substitution of trivalent ions.

**Figure 10.**
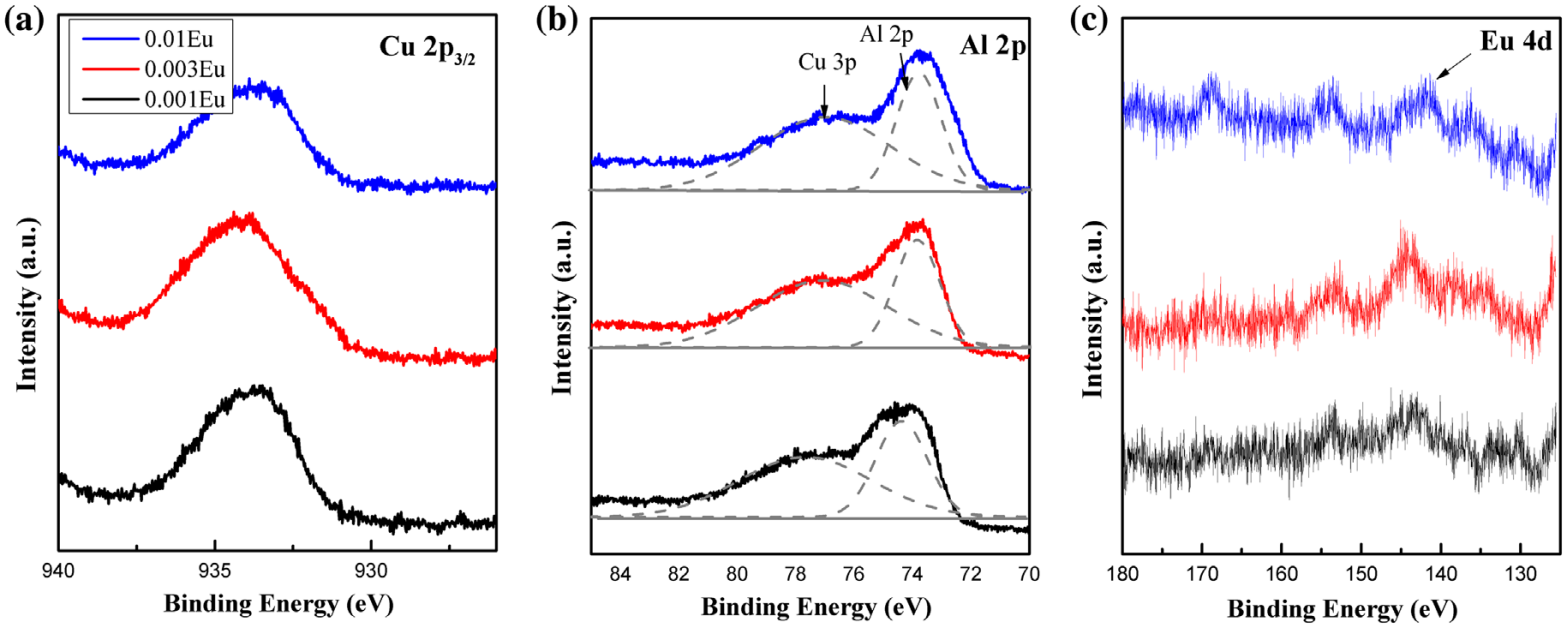
XPS spectra of (a) Cu 2p_3/2_, (b) Al 2p, and (c) Eu 4d levels.

The binding energies (BE) of the atoms were examined by XPS and calibrated with respect to the C 1s BE (284.6 eV). The XPS high resolution Cu 2p, Al 2p and Eu 4d spectra are shown in Figure 10. The BE for Cu 2p_3/2 is_ centered at ~933.4 eV for all three samples with no shift observed with Eu doping. It has been widely acknowledged that in the delafossite structure the strong two-dimensional confinement of Cu-O bonds along the *z*-axis restrains the electronic structure.[6] Therefore the substitution of Eu^3+^ ions along the *ab* plane has little effect on the charge density around Cu^+^ ions. For the spectra shown in Figure 10(b), the peaks can be deconvoluted into Al 2p and Cu 3p components. In contrast to Cu 2p_3/2_ peaks, the Al 2p peaks shift to lower binding energies at higher dopant concentrations (74.52 eV for x = 0.001, 74.09 eV for x = 0.003 and 73.54 eV for x = 0.01) due to the environment change around the MO_6_ octahedra along the *ab* plane. The different Cu 2p_3/2_ and Al 2p BE responses to trivalent ion substitution leads to the conjecture that the introduced rare earth within the MO_2_ layer would insignificantly disturb the hole conduction path in the Cu^+^ layer. Therefore, the p-type conductivity might not be impaired. In Figure 10(c), the presence of Eu 4d spectra indicates the successful doping of europium ions into the delafossite lattice. The trivalent europium compounds with orbit-spin splitting Eu^3+^ 4d_5/2_ and Eu^3+^ 4d_3/2_ have been shown to have a binding energy of 135 and 142.5 eV,[39,40] which is similar to what we observed in our study.

**Figure 11.**
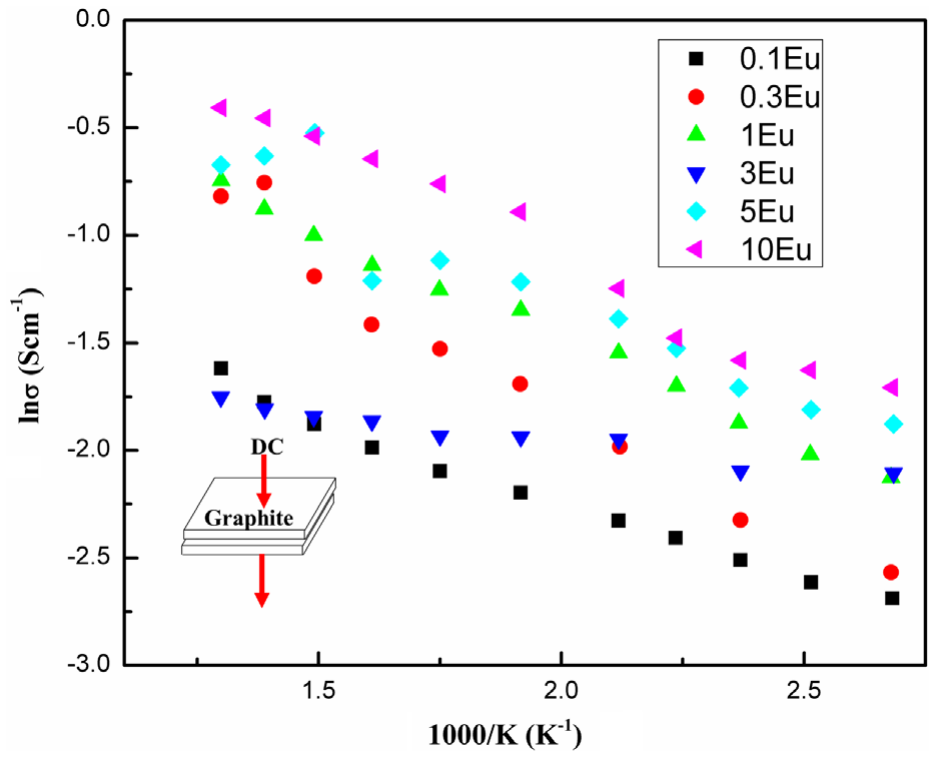
Arrhenius plots of DC electrical conductivity in CuAlO_2_ fibers.

The temperature dependence of electrical conductivity of the electrospun fibers is shown in Figure 11. Arrhenius plots were used to identify the thermal-activation type of these wide band-gap materials. DC current was applied through a piece of fiber mat sandwiched by two rectangular graphite foils, which were used to minimize the contact resistance. The conductivity increases as the temperature increases, showing semiconduction. At ~300 K, the electrical conductivity for 0.1% Eu doped CuAlO_2_ fibers is ~0.05 S cm^−1^ with a thermal activation energy of ~0.09 eV. The room temperature conductivity increased with elevated Eu doping levels, from 0.05 to 0.17 S cm^−1^. This could be attributed to the substitution of Eu cations which induced the lattice distortion and increased the hole concentrations. On the other hand, the substitution of Eu on the Al site may introduce a smaller band-gap associated with CuEuO_2_ and this modification at the near-band-edge may also contribute to the hole conductivity enhancement. Based on the plot shown in Figure 11, when the doping level is as small as 0.1%, logσ decreases linearly with the reciprocal of temperature. However as the doping concentration increased, a variable-range hopping mechanism occurred,[41] which could be seen from the segmented slopes at different temperature ranges. The slope at higher temperature range between 570 and 800 K is lower than that at a lower temperature range between 300 and 400 K. A revisit to PL energy peaks shown Figure 6 could lead to the conjecture that the change in thermal activation may result from the various band-gaps induced by Eu partial substitution, since the band-gaps decrease with the increase of Eu concentration. In addition to the DC temperature-dependent conductivity measurement, the Hall coefficient (R_H_) for the 0.1%Eu doped sample determined by Hall measurement is + 5.48 cm^3^/C, corresponding to a carrier density of 1.14 × 10^18^ cm^−3^, while the heavily doped 10% sample has a RH = +6.94 cm^3^/C and a carrier density of 8.99 × 10^17^ cm^−3^, both showing a p-type semiconductivity.

## 4. Conclusions

CuAl_1-x_Eu_x_O_2_ (x = 0.001, 0.003, 0.01, 0.03, 0.05 and 0.1) fibers have been fabricated through a combination of a chemical solution method and electrospinning technique. This method could effectively synthesize single-phase CuAlO_2_ via one-step annealing in air. The lattice parameter *a* follows a pseudolinear relationship with Eu concentration while the lattice parameter *c* remains almost constant with Eu concentrations. This implied that the delafossite-type CuAlO_2_ could be used as a potential host material for the rare-earth Eu^3+^ partial substitution on trivalent site. Further photoluminescence measurements indicated that upon two excitation wavelengths of 390 and 465 nm, the CuAl_1-x_Eu_x_O_2_ exhibits two emission behaviors from both intrinsic near-band-edge violet emission and Eu^3+ 5^D_0_ → ^7^F_J_ red emission, which shows that this material might be versatile in multi-channel light emitting. All fibers show semiconducting behavior as a function of temperature, and the room temperature conductivity increased monotonically with an increase of Eu concentration. The variation in thermal activation energy as a result of Eu substitution indicates different hopping mechanisms occur at inhomogeneous near-band-edges in the Eu doped delafossite structure.

## Disclosure statement

No potential conflict of interest was reported by the authors.

## References

[CIT0001] Yu R-S, Liang S-C, Lu C-J (2007). Characterization and optoelectronic properties of p-type N-doped CuAlO_2_ films. Appl Phys Lett.

[CIT0002] Ong CH, Gong H (2003). Effects of aluminum on the properties of p-type Cu–Al–O transparent oxide semiconductor prepared by reactive co-sputtering. Thin Solid Films.

[CIT0003] Fang M, He H, Lu B (2011). Optical properties of p-type CuAlO_2_ thin film grown by rf magnetron sputtering. Appl Surf Sci.

[CIT0004] Chen HY, Tsai MW (2011). Delafossite-CuAlO_2_ thin films prepared by thermal annealing. J Nano Res.

[CIT0005] Mo R, Liu Y (2011). Synthesis and properties of delafossite CuAlO_2_ nanowires. J Sol-gel Sci Techn.

[CIT0006] Banerjee AN, Joo SW, Min B-K (2012). Quantum size effect in the photoluminescence properties of p-type semiconducting transparent CuAlO_2_ nanoparticles. J Appl Phys.

[CIT0007] Banerjee A, Chattopadhyay K (2005). Size-dependent optical properties of sputter-deposited nanocrystalline p-type transparent CuAlO_2_ thin films. J Appl Phys.

[CIT0008] Jiang HF, Zhu XB, Lei HC (2011). Effect of Cr doping on the optical–electrical property of CuAlO_2_ thin films derived by chemical solution deposition. Thin Solid Films.

[CIT0009] Tsuboi N, Hoshino T, Ohara H (2005). Control of luminescence and conductivity of delafossite-type CuYO_2_ by substitution of rare earth cation (Eu, Tb) and/or Ca cation for Y cation. J Phys Chem Solids.

[CIT0010] Lee M, Kim T, Kim D (2001). Anisotropic electrical conductivity of delafossite-type CuAlO_2_ laminar crystal. Appl Phys Lett.

[CIT0011] Bu W, Hua Z, Chen H (2005). Epitaxial synthesis of uniform cerium phosphate One-Dimensional nanocable heterostructures with improved luminescence. J Phys Chem B.

[CIT0012] Jia G, Zheng Y, Liu K (2008). Facile surfactant-and template-free synthesis and luminescent properties of one-dimensional Lu_2_O_3_: Eu^3+^ phosphors. J Phys Chem C.

[CIT0013] Yang J, Li C, Cheng Z (2007). Size-tailored synthesis and luminescent properties of one-dimensional Gd2O3:Eu3+ nanorods and microrods. J Phys Chem C.

[CIT0014] Zhang X, Xu S, Han G (2009). Fabrication and photocatalytic activity of TiO_2_ nanofiber membrane. Mater Lett.

[CIT0015] Zhang Z, Li X, Wang C (2009). ZnO hollow nanofibers: fabrication from facile single capillary electrospinning and applications in gas sensors. J Phys Chem C.

[CIT0016] Jacob K, Alcock C (1975). Thermodynamics of CuAlO2 and CuAl2O4 and phase equilibria in the system Cu2O-CuO-Al2O3. J Am Ceram Soc.

[CIT0017] Xiong D, Zeng X, Zhang W (2014). Synthesis and characterization of CuAlO_2_ and AgAlO_2_ delafossite oxides through low-temperature hydrothermal methods. Inorg Chem.

[CIT0018] Liu Y, Olson TL, Wu Y (2014). Luminescence and microstructure of Nd Doped Y_2_Si_2_O_7_ electrospun fibers. J Am Ceram Soc.

[CIT0019] Isawa K, Yaegashi Y, Komatsu M (1997). Synthesis of delafossite-derived phases, RCuO_2+ δ_ with R= Y, La, Pr, Nd, Sm, and Eu, and observation of spin-gap-like behavior. Phys Rev B.

[CIT0020] Chong M, Pita K, Kam C (2004). Photoluminescence of sol–gel-derived Y_2_O_3_: Eu^3+^ thin-film phosphors with Mg^2+^ and Al^3+^ co-doping. Applied Physics A.

[CIT0021] Shahriari DY, Barnabe A, Mason TO (2001). A high-yield hydrothermal preparation of CuAlO_2_. Inorg Chem.

[CIT0022] Tsuboi N, Ohara H, Hoshino T (2005). Luminescence properties of delafossite-type CuYO_2_ doped with calcium, oxygen or rare earth Tb. Jpn J Appl Phys.

[CIT0023] Kumekawa Y, Hirai M, Kobayashi Y (2010). Evaluation of thermodynamic and kinetic stability of CuAlO_2_ and CuGaO_2_. J Therm Anal Calorim.

[CIT0024] Liu Q-J, Zhang N-C, Sun Y-Y (2014). Structural, mechanical, electronic, optical properties and effective masses of CuMO_2_ (M= Sc, Y, La) compounds: first-principles calculations. Solid State Sci.

[CIT0025] Byrne D, Cowley A, Bennett N (2014). The luminescent properties of CuAlO_2_. Journal of Materials Chemistry C.

[CIT0026] Kumar A, Jose R, Fujihara K (2007). Structural and optical properties of electrospun TiO_2_ nanofibers. Chem Mater.

[CIT0027] Scanlon DO, Walsh A, Morgan BJ (2009). Effect of Cr substitution on the electronic structure of CuAl_1− x_Cr_x_O_2_. Phys Rev B.

[CIT0028] Scanlon DO, Watson GW (2010). Conductivity limits in CuAlO_2_ from screened-hybrid density functional theory. J Phys Chem Lett.

[CIT0029] Jacob A, Parent C, Boutinaud P (1997). Luminescent properties of delafossite-type oxides LaCuO_2_ and YCuO_2_. Solid State Commun.

[CIT0030] Naldoni A, Allieta M, Santangelo S (2012). Effect of nature and location of defects on bandgap narrowing in black TiO_2_ nanoparticles. J Am Chem Soc.

[CIT0031] Liu Y, Huang Y, Seo HJ (2014). Blueshift in near-band-edge emission in Y^3+^-doped CuAlO_2_ nanofibers. Opt Mater Express.

[CIT0032] Ahmad A, Jagadale T, Dhas V (2007). Fungus-based synthesis of chemically difficult-to-synthesize multifunctional nanoparticles of CuAlO2. Adv Mater.

[CIT0033] Zhao J, Zhang W, Xie E (2011). Structure and photoluminescence of β-Ga_2_O_3_:Eu^3+^ nanofibers prepared by electrospinning. Appl Surf Sci.

[CIT0034] Chen J, Wang J, Zhang F (2008). Structure and photoluminescence property of Eu-doped SnO_2_ nanocrystalline powders fabricated by sol–gel calcination process. J Phys D Appl Phys.

[CIT0035] Zhang J, Wang Y (2010). Eu^3+^-doped Ba_3_Bi(PO_4_)_3_: A red phosphor for white light-emitting diodes. Electrochem Solid-State Lett.

[CIT0036] Yassin O, Alamri S, Joraid A (2013). Effect of particle size and laser power on the Raman spectra of CuAlO_2_ delafossite nanoparticles. J Phys D Appl Phys.

[CIT0037] Pellicer-Porres J, Martinez-Garcia D, Segura A (2006). Pressure and temperature dependence of the lattice dynamics of CuAlO_2_ investigated by Raman scattering experiments and *ab initio* calculations. Phys Rev B.

[CIT0038] Salke NP, Rao R, Achary S (2012). Raman spectroscopic investigations on delafossite CuLaO_2_ at high pressures. *J* Phys Conf Ser.

[CIT0039] Lu D-Y, Sugano M, Sun X-Y (2005). X-ray photoelectron spectroscopy study on Ba_1−x_Eu_x_TiO_3_. Appl Surf Sci.

[CIT0040] Schneider W-D, Laubschat C, Nowik I (1981). Shake-up excitations and core-hole screening in Eu systems. Phys Rev B.

[CIT0041] Yanagi H, Inoue S-i, Ueda K (2000). Electronic structure and optoelectronic properties of transparent p-type conducting CuAlO_2_. J Appl Phys.

